# A novel methylated cation channel TRPM4 inhibited colorectal cancer metastasis through Ca^2+^/Calpain-mediated proteolysis of FAK and suppression of PI3K/Akt/mTOR signaling pathway

**DOI:** 10.7150/ijbs.70504

**Published:** 2022-09-01

**Authors:** Chan Wang, Jiaxin Chen, Yeye Kuang, Xiaoqing Cheng, Min Deng, Zhinong Jiang, Xiaotong Hu

**Affiliations:** 1 Department of Pathology, Sir Run Run Shaw Hospital, Zhejiang University, Hangzhou 310016, Zhejiang, China.; 2 Key Laboratory of Biotherapy of Zhejiang Province, Sir Run Run Shaw Hospital, Zhejiang University, Hangzhou 310016, China.; 3 Department of Pathology, The First People's Hospital of Fuyang, Hangzhou 311400, China.

**Keywords:** TRPM4, tumor suppressor gene, methylation, metastasis

## Abstract

Colorectal cancer (CRC) is an aggressive malignancy with poor prognosis. It is imperative to elucidate the potential molecular mechanisms that regulate CRC cell aggressiveness. In present study, the transient receptor potential melastatin 4 (TRPM4), a calcium-activated nonselective cation channel, is downregulated in CRC as a novel methylated tumor suppressor gene (TSG). The reduced mRNA level of TRPM4 is due to the epigenetic methylation of its promoter CpG island (CGI). Moreover, ectopic expression of TRPM4 inhibited tumor growth and metastasis both in vitro and in vivo. Our experiments also demonstrate that TRPM4 restructures the CRC cytoskeleton and activates the Ca^2+^-mediated calpain pathway through enhancing calcium influx. The western blot analysis shows that the expression of focal adhesion kinase (FAK), a calpain-mediated proteolytic substrate, is markedly suppressed after ectopic overexpression of TRPM4, besides, Akt (also known as protein kinase B, PKB), phosphatidylinositol 3-kinase (PI3K) as well as its central target mTOR have significantly decreased expression accompanied by elevated E-cadherin and restrained matrix metalloproteinases (MMP2/MMP9) expression. The inhibition of protease calpain effectively relieves the retard of FAK/Akt signals and reverses the migration suppression of TRPM4. Taken together, TRPM4, identified as a novel methylated TSG, employs intracellular Ca^2+^ signals to activate calpain-mediated cleavage of FAK and impede CRC migration and invasion through modulating the PI3K/Akt/mTOR signaling cascade, providing the first evidence that TRPM4 is likely to be a significant biomarker and potential target for CRC therapy.

## Introduction

Colorectal cancer (CRC) is the third most common cancer in the world and has high morbidity and mortality rates. It is frequently diagnosed in the advanced stages with only limited therapeutic strategies available, which made it a major public health problem worldwide 1,2. Multitudes of genetic and epigenetic alternations accumulated gradually in CRC are instrumental in its initiation and progression. Among these changes, inactivation of tumor suppressor genes (TSGs) regulated by epigenetic especially DNA methylation, is of great significance in CRC development 3. It is well established that the hypermethylation of CGIs at promoter regions of TSGs play pivotal role in its disruption and the subsequent tumor progression. Consequently, identification and characterization of novel methylated TSGs is necessary for CRC diagnosis as well as therapy 4,5.

Tumor metastasis is a multi-step pathological process based on a complicated metastatic cascade. The epithelial-mesenchymal transition (EMT), cadherins or integrins-induced loss of intercellular junctions, invasion of extracellular matrix (ECM) as well as the subsequent adhesion and extravasation to remote tissues collectively constitute this cascade 6. Metastatic dissemination is deemed as the main cause of mortality in CRC patients, which heighten the awareness of finding new targets against one or more steps of this process 6,7.

The transient receptor potential (TRP) ion channel proteins are widely expressed in human tissues and consist of six subfamilies including melastatin (TRPM) 8. The nonselective cation channel TRPM4, activated by intracellular Ca^2+^ concentration ([Ca^2+^]i), has a relatively broad pattern of tissue expression and has been found to be upregulated in many human cancers 9,10. However, Sozucan Y et al demonstrated a lower expression level of TRPM4 in CRC tissue in comparison with adjacent normal tissues, suggesting its potential role as TSG 11. Although TRPM4 conducts only monovalent cations such as Na^+^ and K^+^, it plays a central role in controlling intracellular Ca^2+^ concentration ([Ca^2+^]i) through regulating membrane depolarization and shaping other influx driving force such as voltage-dependent calcium channel (VDCC) 9,12,13. Calcium is a remarkably versatile second messenger involved in modulation of various pathophysiological processes 14. In view of its essential role in cytoskeleton rearrangement, cancer metastasis and cell migration, the identification of specific calcium signaling pathway may confer significant clinical benefit.

In present study, we identified TRPM4 as a novel methylated TSG with low expression in CRC by epigenomic study and expression profiling of CRC cell lines. Besides, the function of TRPM4 in CRC progression and the underlying molecular mechanisms have been further explored.

## Materials and Methods

### Public cancer database analysis

The expression analysis of TRPM4 in colorectal cancer was on the basis of data retrieved from TCGA and GEO databases. Level 3 RNA-sequencing data of CRC were downloaded from TCGA data portal (http://portal.gdc.cancer.gov/). GEO datasets were downloaded from GEO database (http://www.ncbi.nlm.nih.gov/geo), including GSE20842, GSE21510, GSE25070, GSE32323, GSE35834, GSE44861 and GSE89076. The statistical analyses were performed by GraphPad Prism software (v6). Kaplan-Meier survival analyses were performed with the Log‐rank test.

### Transcriptome analysis of TRPM4

To investigate the molecular mechanism of aberrant expression of TRPM4 in colorectal cancer, we used online database cBioPortal (www.cbioportal.org) 15, MEXPRESS (http://mexpress.be) 16 to analyze the correlations between mRNA expression of TRPM4 and DNA methylation and copy number variation.

### Cell lines, tumor and normal control tissues

A group of colon cancer cell lines (COLO320, SW620, SW480, LOVO, HCT116, DLD1, SW48, HT29, RKO) was used. The cells were cultured in McCoy's 5A Medium (GE, Healthcare, PAA Laboratories, Pasching, Austria) or Dulbecco's Modified Eagle's Medium (DMEM) (Gibco BRL, Rockville, MD, U.S.) with 10% fetal bovine serum.

125 primary colorectal cancer patients who underwent surgery between February 2004 and June 2006 at the Sir Run Run Shaw Hospital (Hangzhou, Zhejiang, China) were investigated for the immunohistochemistry experiments. Patients in this study had not received preoperative chemotherapy. In addition, we used 10 normal colon mucosa biopsy samples as normal controls and 29 CRC cases for MSP and Western blot. This study was approved by the ethics committee of Sir Run Run Shaw Hospital, Zhejiang University.

### 5-Aza-2'-deoxycytidine (5-Aza) and trichostatin A (TSA) treatment

Cells that do not express TRPM4 were treated with 10μM demethylation agent 5-Aza (Sigma-Aldrich, St Louis, MO) for 72 hours and then further treated with 100 μM TSA (Sigma-Aldrich, St Louis, MO) for 24 hours. Subsequently, cells were collected for RNA and DNA extraction.

### Semi-quantitative RT-PCR

Semi-quantitative RT-PCR was conducted as previously described 17. In the meantime, GAPDH was used as an internal control. Primer sequences used in this study are listed in Supplementary Table 1.

### Bisulfite treatment and promoter methylation analysis

Bisulfite mediation of DNA, methylation specific PCR (MSP) were performed as previously described 17,18. Primers for MSP are listed in Supplementary Table 1.

### TRPM4-expression plasmid and cell transfection

SW48 and HT29 were transfected with control (pCMV6-Entry) or pCMV6-Entry TRPM4 plasmid (Origene, Maryland) using MegaTran 1.0 transfection reagent (Origene, Maryland, U.S.). The stable transfected TRPM4- and mock-expressing clones were selected for further study.

### Colony formation assay

For colony formation assays, 1000 stable transfected and mock cells were plated in a 10cm dish and cultured for 14 to 20 days at 37℃ in 5% CO_2_. Surviving colonies (≥50 cells/colony) were photographed and counted after Giemsa staining. The experiments were carried out in triplicate.

### Transwell cell migration assay

Cells were trypsinized and resuspended in a corresponding medium containing 1% fetal bovine serum at a density of 5×10^5^ cells/ml with 10μg/ml mitomycin C. Then we added one hundred microliters of cell suspension into the upper chamber of a transwell (Corning, NY, U.S.) consisting of polyethylene terephthalate (PET) membranes with 8μm pores. Six hundred microliters medium containing 10% fetal bovine serum was placed in the lower chamber. The chambers were incubated at 37℃ in 5% CO_2_ for 48h and then the cells on the lower chamber was stained with 0.25% crystal violet, and the attached cells were counted and photographed under a microscope. The assay was conducted in triplicate.

### Wound-healing assay

Cell motility was assessed using a wound assay. The SW48 and HT29 cells transfected with TRPM4 and control were cultured in 6-well dishes with 10μg/ml mitomycin C (MCE, NJ, USA). The cell layers were wounded with sterile tips and washed with phosphate-buffered saline (PBS). Cells were cultured in fresh medium and photographed under a phase contrast microscope at different time after wounding. Each assay was conducted in triplicate.

### Immunofluorescence assay

Cells were seeded on glass slide in 6-well dishes. The next day, these cells were washed three times with PBS. Thereafter, the cells were fixed and permeabilized by sequential incubation in 4% p-formaldehyde for 20 min at room temperature and in 0.1% Triton X-100 for 10min, followed by 30 min incubation in a blocking buffer (3% BSA). The actin cytoskeleton was visualized with a Rhodamine-phalloidin (5U/ml, Invitrogen) which need to incubate for 30 min. The nuclear were stained with DAPI for 20 min. For the detection of proteins, after being blocked by Albumin Bovine V (Biosharp), samples were incubated with primary antibody to FAK (1:200) at 4℃ overnight following secondary-antibody FITC (Beyotime). The nuclear were stained with DAPI for 20min. Photomicrographs were obtained using a fluorescence microscope.

### Measurement of intracellular Ca^2+^ concentration

We used Fluo-3 AM (Beyotime) to detect the change of the intracellular Ca^2+^ level 19. Cells were loaded with 2μM Fluo-3 AM diluted in Krebs-Ringer buffer [KRB; 1.5 mM NaH_2_PO_4_ (pH=7.4), 0.7 mM Na_2_HPO_4_, 4.5 mM KCl,10 mM d-glucose, 0.5 mM MgCl_2_, 120 mM NaCl] for 30 min in a CO_2_ incubator. After washing with KRB to remove the residual dye, the cells were harvested, washed with Ca^2+^-free PBS and analyzed by flow cytometry. To determine the source of increased [Ca^2+^]i, the pre-treatment of cells was performed using 10 mM EGTA, 10 μM BAPTA-AM+2 mM CaCl_2_ and 2mM CaCl_2_ for 30 min, respectively. Then washed with Ca^2+^-free PBS and analyzed by flow cytometry.

### Calpain activity assay

We used the cell-permeable fluorogenic calpain substrate, S-LIVY-AMC to measure calpain activity. Cells were harvested and then washed three times with HEPES-buffered Hank's balanced salt solution (HBSS; pH=7.4) without phenol red, then resuspended in HEPES-HBSS at 3×10^5^ cells/ml, and preheated for 10 min at 37℃ in a CO_2_ incubator. Next, the substrate S-LLVY-AMC (25 μM) was added and the AMC fluorescence intensity was measured using a spectrofluorometer (BIO-TEK ELX800) at excitation and emission wavelengths of 400 and 505 nm, respectively.

### Immunohistochemistry

The ChemMateEnVision Detection Kit (DAKO, Carpinteria, CA, U.S.) was used for IHC staining. The sections were incubated with the TRPM4 antibody (1:100 dilution; HPA042, Sigma, CA, U.S.) overnight at 4℃. Then the sections were incubated with the ChemMateEnVision/HRP, Rabbit/Mouse reagent for 30 min. At last, the sections were stained with ChemMate DAB+ chromogen within the kit. The slides were lightly counterstained with hematoxylin.

The expression level of TRPM4 in tumor tissues was scored according to the intensity of cytoplasmic staining, the percentage of positive cells and subcellular localization. Patients were divided into two groups including high TRPM4 expression and low TRPM4 expression to check the correlation between TRPM4 expression level and clinicopathological characteristics. Immunostaining was scored independently on separate occasions by two investigators who were blinded to the patient-related clinical information.

### Protein extraction and Western blot analysis

Cells were harvested and lysed in a RIPA lysis buffer (Beyotime, Hangzhou, Zhejiang, China) added with proteases inhibitors. We used a BCA Protein Assay Kit to deternine the protein concentration. Cell lysates (30 ug protein/line) were separated on 8%-12% sodium dodecylsulfate polyacrylamide gel electrophoresis (SDS-PAGE) gel for nitrocellulose membrane blotting (Bio-Rad, Hercules, CA, U.S.). The blotted membranes were blocked in 5% skim milk at room temperature for 1h and incubated with primary antibodies (dilution 1:1000) overnight. Afterwards, the blotted membranes were incubated with secondary antibodies (1:2000) in TBST for 1h. Primary and secondary antibodies were purchased from Cell Signaling Technologies (Beverly, MA, U.S.). Detection was carried out using the ECL kit (Pierce Chemical Co., Rockford, IL). Finally, the blots were developed using a Fujifilm Las-4000 Imaging System.

### Tumor growth and metastasis in nude mice

The in vivo experiments were approved by the animal care committee of Sir Run Run Shaw Hospital, Zhejiang University. HT29 cells transfected with TRPM4 and mock (3×10^5^ cells in 0.1ml PBS) were injected subcutaneously into the right dorsal flanks of female BCLB/c nude mice (6-week-old, 5 mice per group). Tumor volume was measured every 2 days for 4 weeks. We used the following formula: ((short diameter)^2^×(long diameter)/2 to calculate the tumor volume.

For the peritoneal metastasis model, the female nude mice were randomly divided into two groups (6-week-old, 8 mice per group). HT29 cells transfected with TRPM4 and Mock (3×10^6^ cells in 0.1ml PBS) were intraperitoneally injected into nude mice. Six weeks after injection, the mice were killed. We determined the presence or absence of any bloody ascites, and the liver metastasis of tumor cells. The liver tissues were fixed with 4% paraformaldehyde, embedded in paraffin and cut into 5 μm sections and then subjected to hematoxylin-eosin staining.

For the liver metastasis model, 5 nude female mice (6-week-old) were included in each group. Mice were anaesthetized with pentobarbital sodium (50 mg/kg) by intraperitoneal injection, then the mice were fixed on the operating table. A 0.5cm incision was made in the left flank and the spleen was separated and exposed, then, 5×10^6^ cells suspended in 50 μL PBS were injected slowly into the lower pole of the spleen, and an alcohol wipe was pressed on the injection site for 1 min. The spleen was returned to the abdominal cavity, and the incision was sutured after no bleeding was found. After 6 weeks, the mice were killed to examine the liver metastases of tumor cells. Liver metastases were confirmed by HE staining. Furthermore, we also counted the numbers of metastatic tumor nodules in the liver and calculated the percentage of tumor area to the entire liver for each mouse. Tumor surface area was measured in pixels by tracing all the tumor borders and the whole liver surface with image processing software (ImageJ, version 1.52a, http://imagej.nih.gov/ij/download.html).

### Statistical analysis

Statistical analysis was performed in SPSS 22.0 for Mac (SPSS lnc., Chicago, IL, U.S.). Results presented in the figures are expressed as values of mean± standard deviation (s.d.). We used the two tiled Chi-square test to analyze the relation between TRPM4 expression and clinicopathological parameters. Survival curves were constructed using the Kaplan-Meier method. Differences were considered significant when the associated p-value was less than 0.05.

## Results

### Downregulation and methylation of TRPM4 in Public cancer database

**Figure 1:**
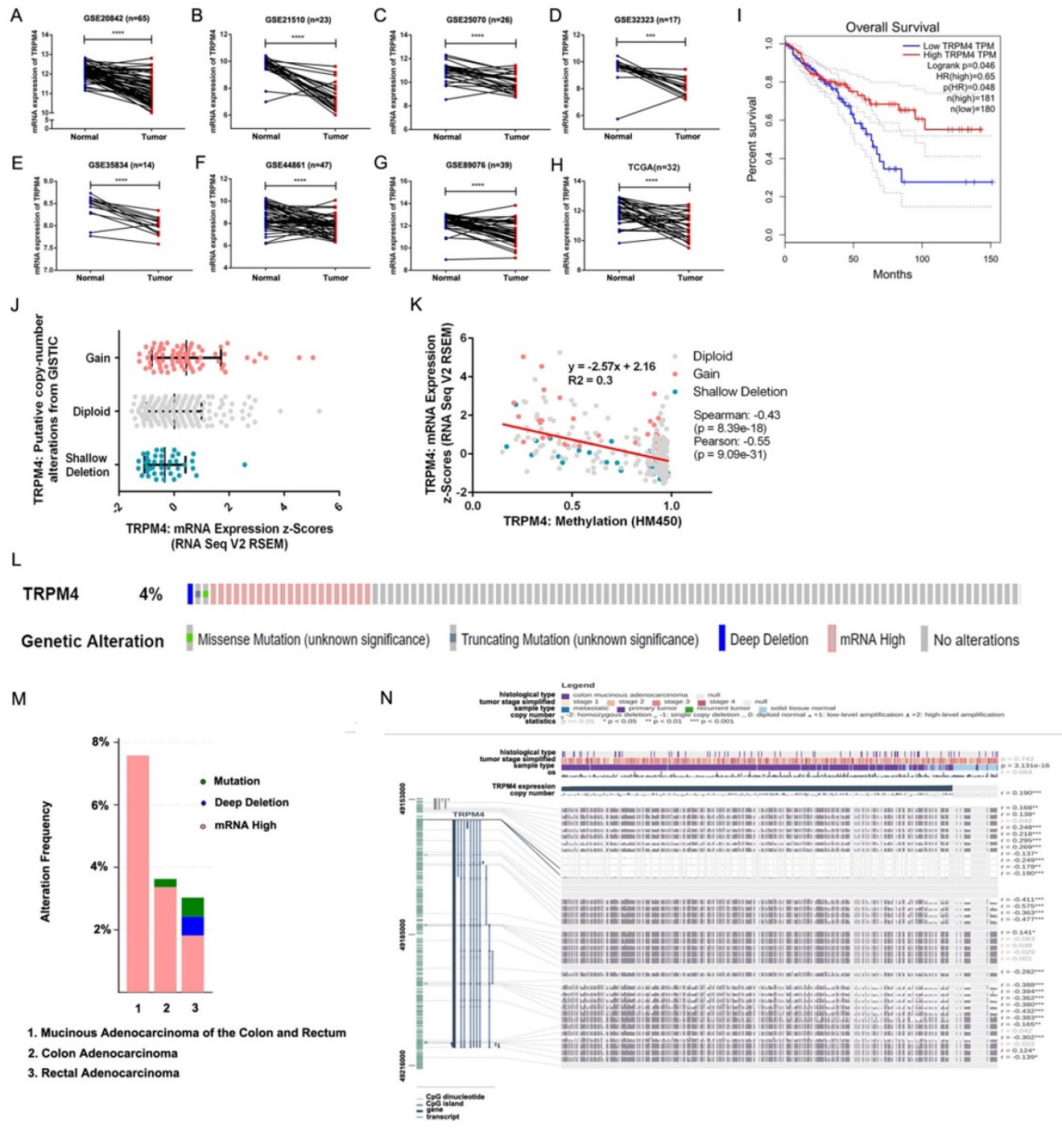
** TRPM4 mRNA expression is downregulated in CRC by promoter methylation. (A-H)** Expression of TRPM4 was frequently downregulated in CRC tumor tissues compared with normal tissues in public databases.** (I)** Lower TRPM4 expression shows shorter overall survival. **(J, K)** Relationship between TRPM4 mRNA level and promoter methylationand copy number alteration in CRC (cBioPortal). **(L, M)** OncoPrint of TRPM4 alterations in CRC. **(N)** TRPM4 expression and promoter methylation are negatively correlated at the overall level, which is confirmed by the Pearson correlation coefficients on the right (MEXPRESS). Data are shown as mean±SD *p < 0.05; **p < 0.01; ***p < 0.001, ****p < 0.0001

The expression level of TRPM4 in the matched-paired CRC and normal tissue samples was investigated in the samples from TCGA and GEO datasets. We found that TRPM4 was notable downregulated in tumor tissues in these datasets (Figure 1A-H). Lower TRPM4 expression shows shorter overall survival (Figure 1I). To investigate the molecular mechanism by which TRPM4 expression is reduced in CRC, we analyzed the types and frequency of TRPM4 mutation in CRCs from TCGA and the relationship between its expression and DNA methylation and copy number variance by cBioPortal. The results showed a modest increase of TRPM4 expression as the increase of the copy number (Figure 1J). However, the expression of TRPM4 negatively correlated with DNA methylation (Spearman: -0.43, p=8.39e-18) (Figure 1K). The data of the TCGA database showed genetic alteration of TRPM4 in 24 of 636 CRC samples (3.77%) (Figure. 1L). These alterations included deep deletion in one case (0.16%), mRNA upregulation in 21 cases (3.30%), missense mutation in one case (0.16%) and truncating mutation in one case (0.16%). The pattern of these transcriptional changes of TRPM4 among various CRC subtypes is shown in Figure 1M.

Next, we used MEXPRESS to analyze the correlation between expression level of TRPM4 and DNA methylation of CpG islands. The samples shown in Figure 1N are ordered by expression value. There was a negative correlation between the expression of TRPM4 in COAD and the level of DNA methylation in the promoter at the overall level, which was confirmed by Pearson correlation coefficients (r up to -0.575, p<0.001). The result of MEXPRESS analysis also showed that the mRNA expression level of TRPM4 was much lower in colon cancer samples than those in control samples (p=3.13e-16). Therefore, these data demonstrated that the promoter DNA hypermethylation of TRPM4 may lead to downregulation of its mRNA expression.

### Downregulation and methylation of TRPM4 in CRC cell lines

**Figure 2:**
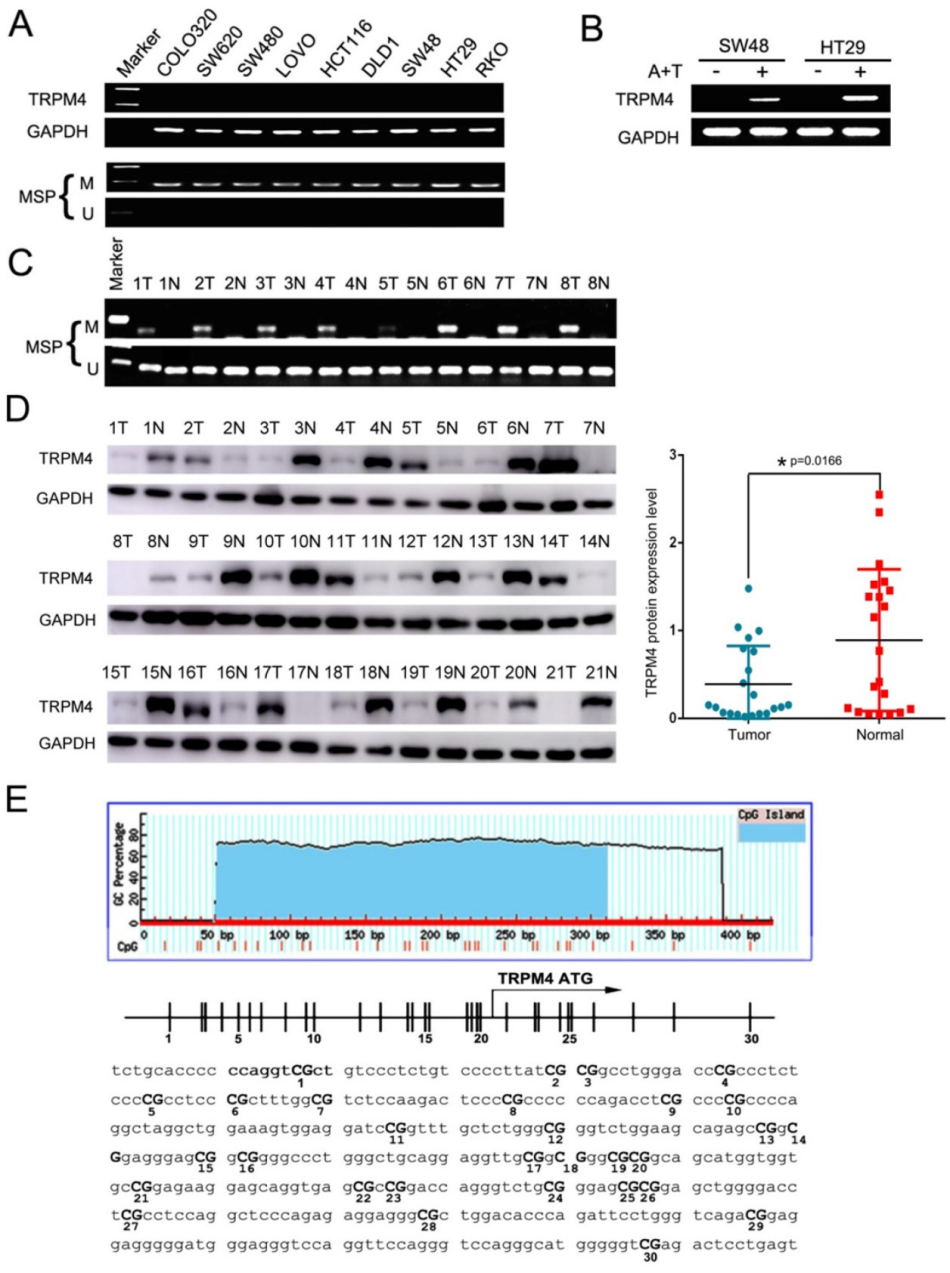
** Analysis of expression level and CGI methylation of TRPM4 in CRC cells. (A)** Decreasing and silencing of TRPM4 in CRC cell lines due to the CGI methylation of its promoter, with GAPDH as controls. **(B)** Pharmacological demethylation with 5-Aza and TSA recovered TRPM4 expression in methylated cell lines. Representative results are shown. A+T: treatment with 5-Aza and TSA. **(C)** Representative analysis of TRPM4 methylation in primary tumors (T) and corresponding noncancerous tissues (N) by MSP. **(D)** TRPM4 protein expression level in 21 paired CRC samples examined by Western Blot. **(E)** The sequence of TRPM4 promoter region, vertical lines indicate individual CpG sites. M methylated; U unmethylated.

First, TRPM4 mRNA expression was examined in CRC cell lines by semi-quantitative RT-PCR, the results showed silenced expression in most of the cell lines (Figure 2A). Analysis of whole-genome CpG methylation profiles (methylomes) revealed that there was a typically CpG island (CGI) in TRPM4 promoter region (Figure 2E). Therefore, we then examined its methylation status by methylation-specific PCR (MSP) and as a result, TRPM4 CGI was methylated in all cell lines with silenced or reduced expression (Figure 2A). Futhermore, after the treatment of demethylation by TSA and 5-Aza in methylated and silenced cell lines, TRPM4 expression level was significantly restored. The representative results are shown in Figure 2B. We next determined TRPM4 methylation in 8 primary CRC tissues as well as pair-matched normal tissue by MSP and found that its methylation levels in tumor tissues were higher than non-tumor tissues in 8 cases (Figure 2C). Next, we examined TRPM4 protein expression in 21 primary CRC tissues as well as pair-matched normal tissue by Western Blot. The results showed that 14 of 21 pairs (66.7%) demonstrated reduced TRPM4 protein expression in the tumor compared with the adjacent normal tissue (Figure 2D). These results suggested that the transcription of TRPM4 is regulated by promoter CpG methylation (Figure 2E).

### Correlation of TRPM4 expression with clinicopathological characteristics of CRC patients

**Figure 3:**
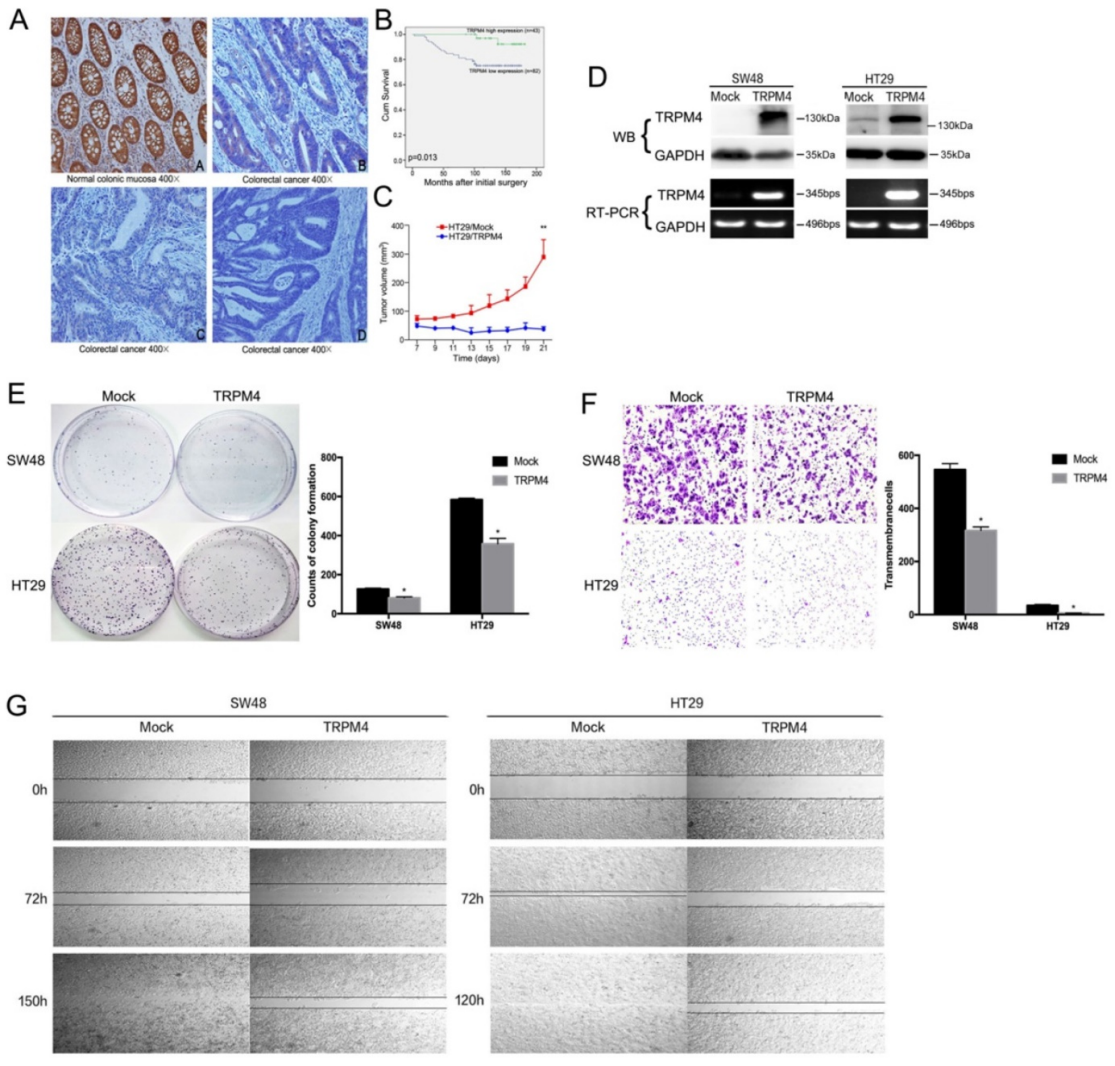
** Efforts of TRPM4 overexpression on tumor cell growth and migration and survival analysis. (A)** Representative immunohistochemical staining for TRPM4. TRPM4 was nearly silenced in tumor tissues, while it was highly expressed mainly in the cytoplasm of paired non-tumor tissues. Original magnification: 400×. **(B)** Overall survival analysis results in CRC patients. **(C)** TRPM4 inhibited tumor growth in nude mice. Results of tumor growth in nude mice inoculated subcutaneously with HT29/Mock and HT29/TRPM4 cells. *: p<0.05. **(D)** TRPM4 expression in stably transfected cells verified by RT-PCR and Western blot. **(E)** Representative colony formation assay and quantitative analysis.** (F)** Migration assay and quantitative analysis. **(G)** Wound-healing assay. Original magnification: 100×. All values are the mean ± s.d. of three independent experiments. *: p<0.05.

TRPM4 protein expression was also investigated in 125 primary CRC tissues and 10 normal colonic mucosa biopsy samples by immunohistochemistry. Representative immunohistochemical staining results showed in Figure 3A manifested that the expression level of TRPM4 was silenced or markedly weak in most primary tumor tissues with only 34.4% (43/125) of them showed high expression. However, TRPM4 exhibited high expression in the cytoplasm of epithelial cells in all normal colonic mucosa.

The correlation between TRPM4 expression and clinicopathological features of CRC patients are showed in Table 1. Low expression of TRPM4 was strongly correlated with invasion (p<0.001), more lymph node metastases (p=0.008), high clinical stage (p<0.001), more distant metastases (p=0.025). Intriguingly, Kaplan-Meier survival analysis indicated that the overall survival rate was considerably higher in patients with TRPM4 overexpression versus those with lower expression, suggesting the strong relationship between TRPM4 expression and CRC prognosis (Figure 3B).

### TRPM4 inhibits cell proliferation and migration of CRC

The clinicopathological parameters and the silencing of TRPM4 by promoter methylation in CRC indicates that TRPM4 is probably a tumor suppressor. Then we studied the effort of ectopic expression of TRPM4 on CRC migration and growth. TRPM4 expression plasmid was stably transfected into SW48 and HT29 cell lines with complete methylation and silenced expression of TRPM4. The forced expression of TRPM4 was verified by RT-PCR and western blot (Figure 3D). We used colony formation assays to see whether the aberrant expression of TRPM4 was responsible for CRC growth, the results showed that the efficiency of colony formation of transfected cells in plate was significantly suppressed compared with vector-transfected mock cells (Figure 3E, p<0.05). These demonstrate the proliferation inhibitory function of TRPM4 in virto. In order to assess whether overexpression of TRPM4 was instrumental in CRC migration and invasion, wound-healing assays and transwell migration were performed and showed that TRPM4 also possesses inhibitory activity of tumor migration and invasion (Figure 3F, 3G, p<0.05). All results above indicated that TRPM4 acts as a tumor suppressor in CRC via impeding cell proliferation as well as metastasis.

### TRPM4 reorganizes cytoskeleton and inhibits tumor growth in nude mice

**Figure 4:**
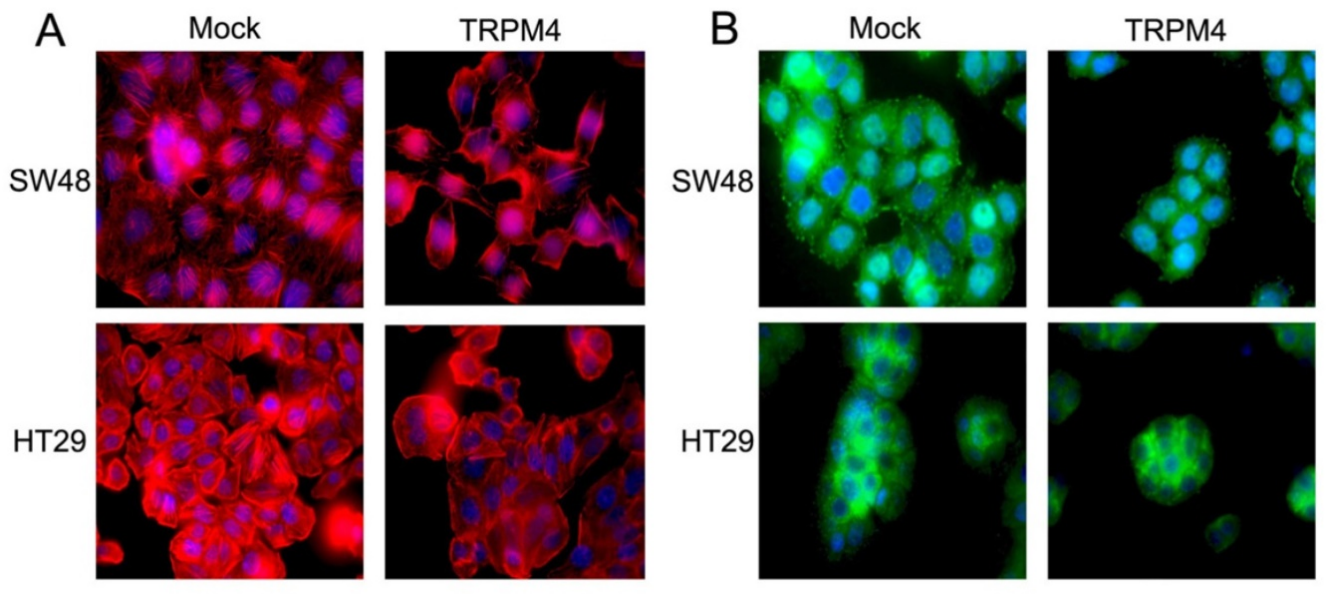
** Representative immunofluorescence images of actin cytoskeleton and FAK. (A)** Representative immunofluorescence images of actin cytoskeleton system destruction in SW48 and HT29 cells after ectopic TRPM4 expression. Original magnification: 1000×.** (B)** Representative immunofluorescence staining of FAK in SW48 and HT29 cells overexpressing TRPM4 with controls. Original magnification: 1000×.

According to the above results, we hypothesized that TRPM4 expression correlative with cytoskeleton system. The actin microfilaments in SW48 and HT29 cells were detected by immunofluorescence. The number of microfilaments was significantly reduced after TRPM4 expression in comparison with mock cells, revealing that TRPM4 is likely to remodel cytoskeleton of CRC (Figure 4A). We further evaluated the efforts of TRPM4 on tumor growth of HT29 cells in nude mice in vivo (n=5, respectively). As the growth curve shown in Figure 3C, the tumor size in TRPM4-transfected nude mice was markedly smaller than that in vector-transfected one, with hardly any tumor growth. This result suggests that TRPM4 inhibits tumor growth in vivo.

In a summary, these results suggest TRPM4 may regulate tumor cell migration through cytoskeleton restructure and suppresses CRC carcinogenesis in vivo.

### TRPM4 inhibits tumor metastasis in nude mice

**Figure 5:**
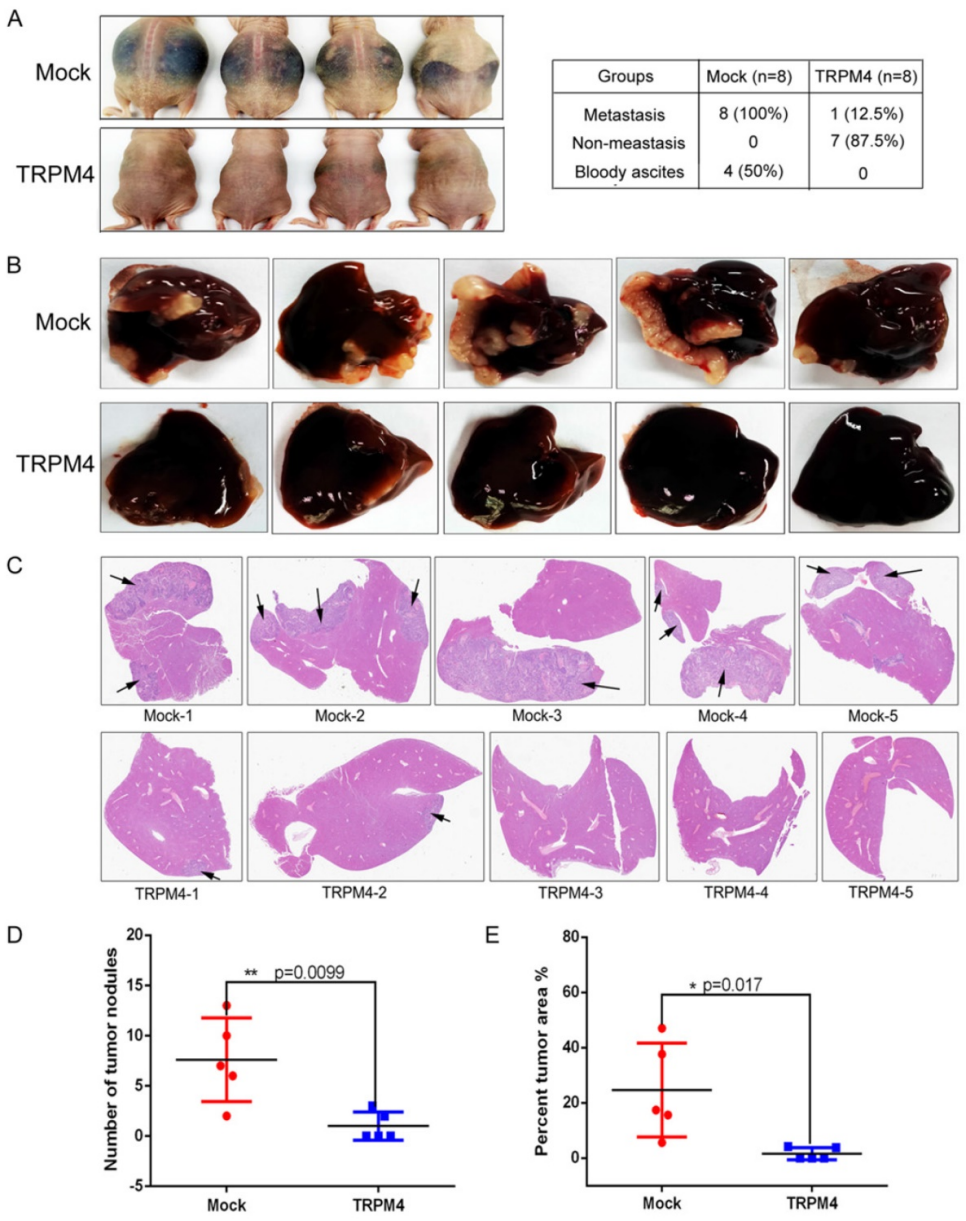
** TRPM4 inhibited tumor metastasis in vivo. (A)** Representative bloody ascites images of metastasis mice and statistical analysis of metastasis in the two groups of nude mice intraperitoneally injected with HT29/Mock and HT29/TRPM4 cells. **(B)** Images of liver metastases of nude mice injected HT29/Mock and HT29/TRPM4 cells in spleen.** (C)** Liver cross sections stained with H&E of nude mice injected HT29/Mock and HT29/TRPM4 cells in spleen. More liver tissue was inundated with metastase in vector-transfected group.** (D)** Scatter diagram showed the number of metastatic nodules was more in nude mice injected with HT29/Mock cells in spleen. **(E)** Percentage of tumor area to total liver area was higher in nude mice injected with HT29/Mock cells in spleen compared with HT29/TRPM4 group. The results were represented as mean ± SD, * p<0.05,**P < 0.01.

To explore the correlation between TRPM4 and the metastasis of CRC in vivo, we next used two experimental metastasis mouse models to investigate the effects of TRPM4 on metastasis: (1) implantation of cells into the abdominal cavity of female nude mice by intraperitoneal injection; (2) injection of cells into the spleen of female nude mice. Four of eight mice intraperitoneal injected with vector-transfected HT29 cells developed peritoneal metastasis with large volumes of bloody ascites. But none of the mice injected with TRPM4-transfected HT29 cells had ascites. Representative figures are shown in Figure 5A. H&E staining was performed to confirm the presence of liver metastasis (Supplementary Figure 1). Although there were no obvious nodules in the liver surface of all mice, microscopic analysis of H&E staining liver sections showed that there were micro-metastasis in all eight mice injected with vector-transfected HT29 cells, but only one mouse injected with TRPM4-transfected HT29 cells had micro-metastasis in liver sections.

In addition, we generated another liver metastasis model by injecting CRC cells into the spleen of nude mice to determine again whether TRPM4 repressed CRC metastasis in vivo. Results revealed that more metastatic nodules were found in vector-transfected HT29 cells group compared with TRPM4-transfected HT29 cells group (Figure 5B, 5D). Furthermore, we also calculated the percentage of tumor area to the entire liver for each mouse (Figure 5E). Compared with TRPM4-transfected group, the liver tumor area was larger in the vector-transfected HT29 cells group. H&E staining of the liver further confirmed that more liver tissue was inundated with metastase in vector-transfected group compared with TRPM4-transfected group (Figure 5C). The amplified pictures of H&E staining of liver tissue sections were showed in Supplementary Figure 2. These results indicate that TRPM4 really inhibit colorectal cancer liver metastasis in vivo effectively.

### TRPM4 enhances intracellular Ca^2+^ level by inducing calcium influx and promotes calpain activation

**Figure 6:**
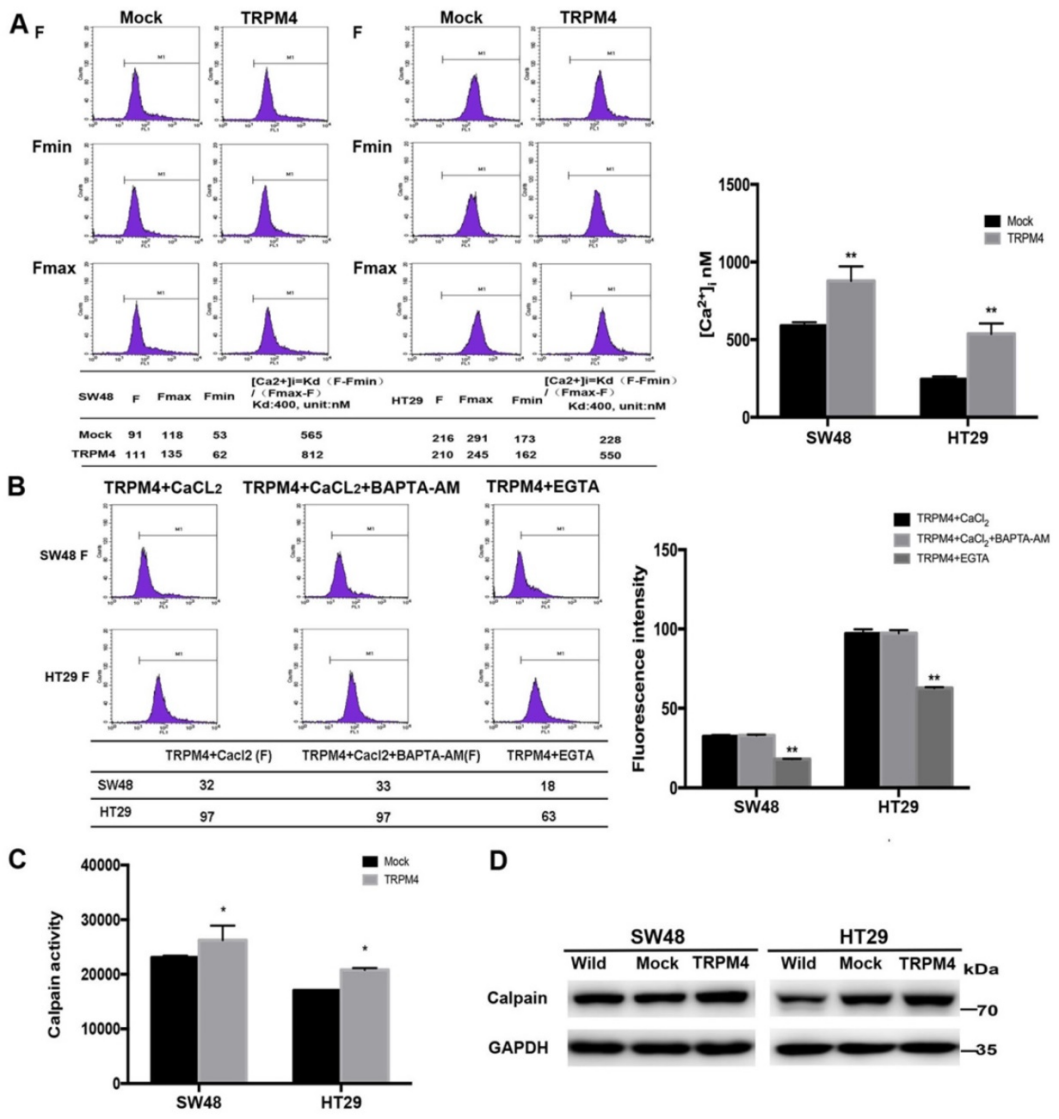
** Efforts of ectopic TRPM4 expression on the level of cytosolic calcium concentration, calpain activity and the remodeling of cytoskeleton. (A)** TRPM4 expression remarkably increased the level of cytosolic Ca^2+^. **(B)** The [Ca^2+^]i level was evidently decreased after EGTA treatment compared with CaCl_2_-treatment and BAPTA-AM+CaCl_2_-treatment control groups. **(C)** TRPM4 expression enhanced the calpain activity. (D) Expression of calpain examined by western blot.

TRPM4 is able to regulate intracellular Ca^2+^ level through membrane depolarization and the inhibitory function of TRPM4 in CRC metastasis has been validated by previous studies. Since the strong relation between calcium and tumor migration, we studied the effort of TRPM4 expression on cytosolic Ca^2+^ levels. TRPM4 expression dramatically enhanced the cytosolic calcium level compared with controls (Figure 6A). Increased intracellular Ca^2+^ concentration ([Ca^2+^]i) can result from extracellular influx or intracellular liberation, mainly from the endoplasmic reticulum (ER). To delineate the exact source of TRPM4-induced [Ca^2+^]i increase, we tested TRPM4-transfected SW48 and HT29 cells in a Ca^2+^-free solution (cells treated with extracellular Ca^2+^ chelator EGTA) and Ca^2+^-containing solutions (cells treated with intracellular chelator BAPTA-AM and CaCl_2_), both of which were compared with cell group that only treated with CaCl_2_ to simulate the physical condition in human bodies. As shown in Figure 6B, Ca^2+^elevation in EGTA treatment cell lines was almost half less than that in Ca^2+^-containing solutions while there was no obvious difference after BAPTA-AM and CaCl_2_ treatment, which revealed that TRPM4-induced [Ca^2+^]i elevation is mainly caused by calcium influx from the extracellular space.

The calcium-dependent protease calpain has been validated to participate in various pathophysiological processes involving migration and cytoskeletal reorganization 20. To further examine the Ca^2+^-mediated signaling pathway, we investigated whether TRPM4 expression activates calpain. Results showed that calpain activity was significantly higher in TRPM4-transfected cells compared with controls (Figure 6C). The western blot analysis also found enhanced expression of calpain after TRPM4 transfection (Figure 6D). These results indicated that TRPM4 increases cytosolic Ca^2+^ level through calcium influx and induce calpain activation in CRC cells.

### TRPM4 induces calpain-mediated FAK proteolysis and restraint of PI3K/Akt/mTOR signaling pathway

**Figure 7:**
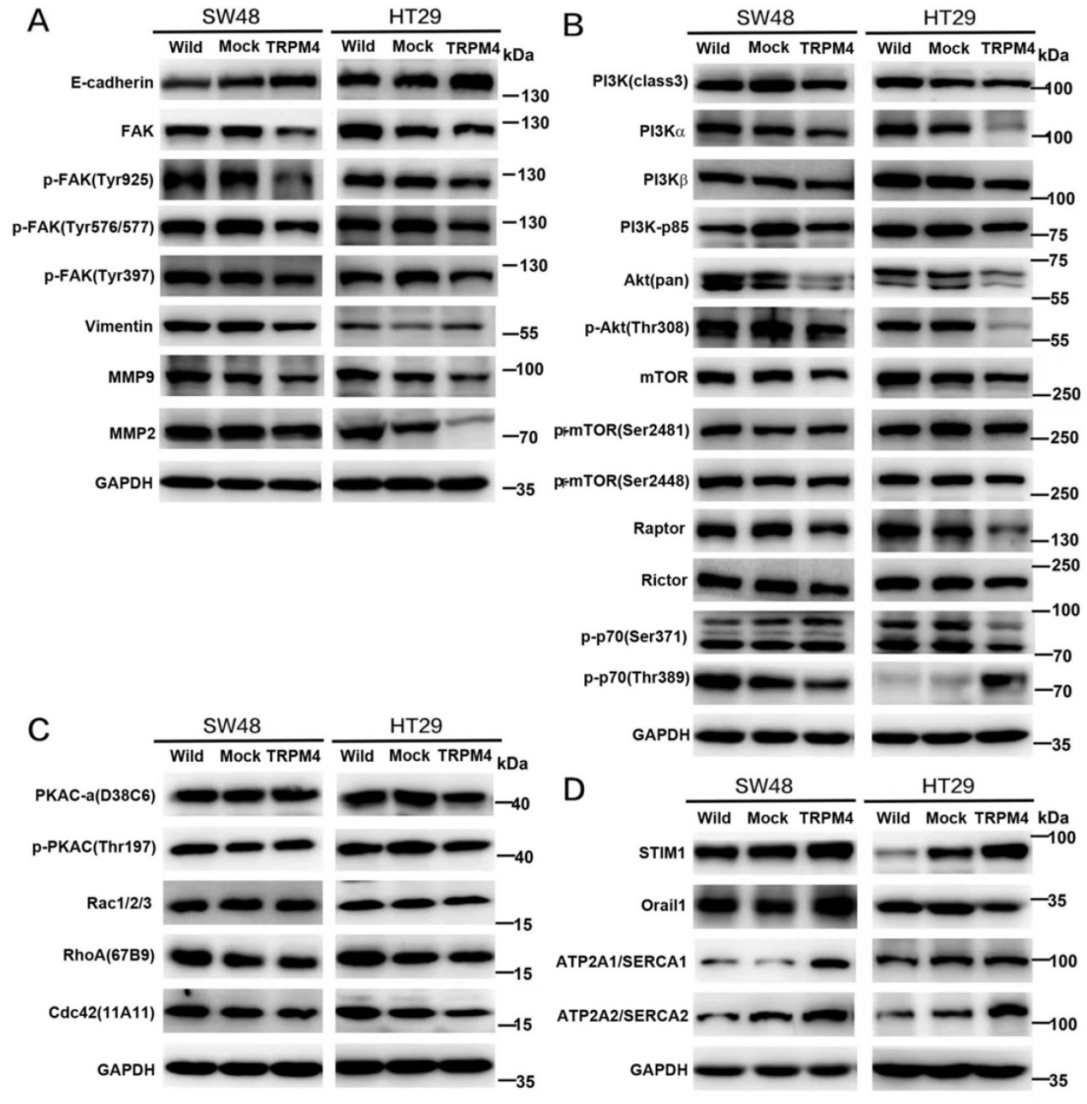
** Efforts of ectopic TRPM4 expression on calpain substrates, cell adhesion, calcium channels and migration-related proteins expression. (A)** TRPM4 significantly inhibited FAK expression and changed the key protein expression level in tumor metastasis. **(B)** TRPM4 expression significantly impeded the PI3K/Akt/mTOR signaling pathway. **(C)** Expression of several Rho family small GTPases examined by western blot analysis in TRPM4-tranfectd cells and controls. **(D)** Ectopic TRPM4 expression activated SOCE and PMCA-related proteins.

Above results have validated that TRPM4 expression plays an important role in tumor migratory and cytoskeletal regulation as well as Ca^2+^-calpain activation. In view of that calpain localized to focal adhesion has potential function of focal adhesion proteins (FAs) cleavage, we checked FAs by Western blot and found both phospho-FAK and total-FAK protein contents were dramatically lower in TRPM4-expressing SW48 and HT29 cells than corresponding controls. Another calpain substrate termed vimentin was also be inhibited by TRPM4 (Figure 7A). Besides, to further examine the expression level of FAK, we processed immunofluorescence assay. As results shown in Figure 4B, the FAK fluorescence particles was significantly decreased in TRPM4-transfected cell lines than mock controls. We also detected some vital proteins and protein kinases which play key roles not only in controlling tumor migration and invasion but also in FAK downstream pathways (Figure 7A, 7B). TRPM4 expression contributed considerably to suppress both total and phosphorylated PI3K and Akt, the main downstream target mTOR was also inhibited. In addition, the expression of E-cadherin, the central component of adhesion junctions, was upregulated while matrix metalloproteinases (MMP2 and MMP9) which are capable of degrading and shedding several extracellular matrix (ECM) components were downregulated, relative to controls. Some key components of calcium-related channels on plasma membrane especially store operated calcium channels (SOC) were examined and we found that the expression of both calcium release-activated calcium modular 1 (Orai1) and stromal interacting molecule 1 (STIM1) were increased. Intriguingly, the participants of plasma membrane Ca^2+^-ATPase (PMCA) termed ATP2A1 and ATP2A2 showed improved level after ectopic TRPM4 expression (Figure 7D). Besides, we checked some main proteins of Rho family small GTPases and found decreased expression of Rac1/2/3 and cdc42 (Figure 7C). These results suggest that TRPM4 retards PI3K/Akt/mTOR signaling pathway induced by Ca^2+^-calpain cleavage of FAK in CRC to impede tumor migration and invasion.

### The cleavage of FAK and suppression of Akt pathway can be reversed by calpain inhibitor

**Figure 8:**
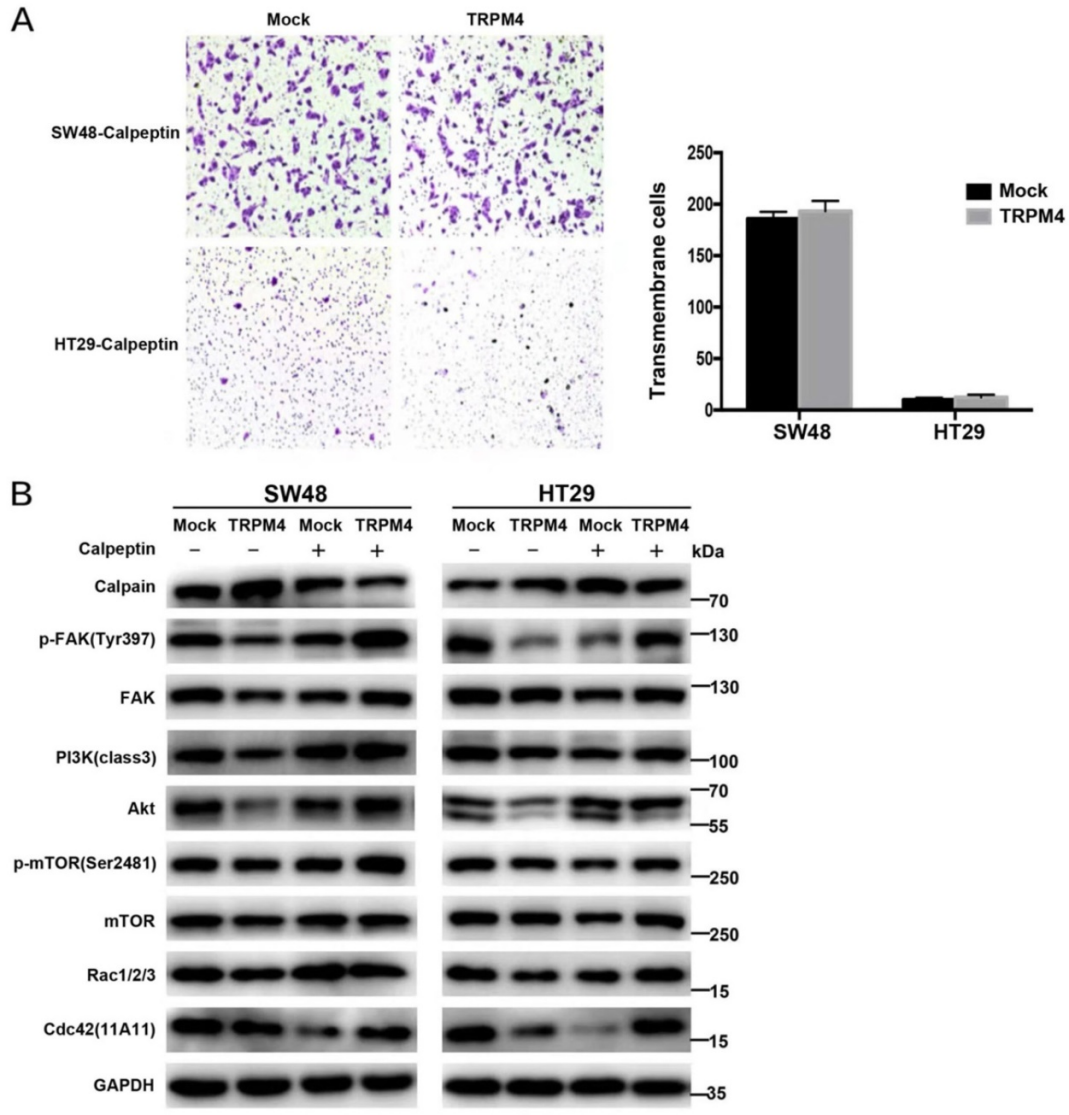
** Efforts of Calpeptin treatment on cell migration and the expression of main proteins in FAK/Akt signaling pathway. (A)** Migration assay and quantitative analysis. **(B)** Expression of several key components in FAK/Akt pathway examined by western blot analysis in TRPM4-tranfectd cells and controls after calpeptin treatment.

Since FAK is one of the well-known substrates of calpain and PI3K/Akt signaling is recognized as canonical target pathway of FAK 20, whether the inhibition of calpain can rescue the suppressive influence of TRPM4 overexpression on downstream key molecules and remove the migration inhibitory function of TRPM4 is well worth investigating. We treated TRPM4-transfected SW48 and HT29 cells with calpain inhibitor calpeptin and used transwell assay and Western blot analysis to further examine the role of calpain (Figure 8A, 8B). The results of transwell assay showed that the migration suppression of TRPM4 was reversed after calpeptin treatment. Additionally, calpeptin rescued the suppression of TRPM4 on both total and phosphorylated FAK along with PI3K/Akt/mTOR signaling cascade according to Western blot analysis. The expression of Rho family small GTPases Rac and cdc42 were also increased after calpeptin treatment. These results further proved the central role of calpain in CRC metastatic regulation.

## Discussion

The cation channels of transient receptor potential (TRP) superfamily are ubiquitously expressed in human body and display diverse functions on various physiological and pathological conditions 21. Based on sequence homology and channel function, they can be divided into 7 subfamilies including TRPP, TRPM, TRPV, TRPA, TRPC, TRPN and TRPML. In recent years, the relationship between TRP channels and cancer has been increasingly clarified 21,22.

The function of TRPM subfamily members are much less studied than those of the TRPV and TRPC family. TRPM4, an intracellular Ca^2+^-activated but Ca^2+^-impermeable voltage-dependent cation channel, has 2 splicing variants, TRPM4a and TRPM4b but only TRPM4b is a widely expressed functional spliceosome which is designated as TRPM4 in research studies 23,24. Depend on Ca^2+^-activated phosphatidylinositol 4,5-bisphosphate (PtdIns(4,5)P2) and phospholipase C (PLC), TRPM4 activation is able to activate VDCC or soltage-gated sodium channels due to the depolarization of plasma membrane potential, which endow TRPM4 the potential to elevate Ca^2+^ influx 25. There have been reports indicate that TRPM4 could be modulated by oxidative stress and result in numerous human diseases such as vascular, neurodegenerative or cardiac diseases 8. Overexpression of TRPM4 endowed cells with higher susceptibility to adenosine triphosphate (ATP) depletion or oxidative stress induced accidental necrosis 26. To date, TRPM4 has been found to be upregulated in various cancers. For instance, it is responsible for prostate cancer migration and proliferation and can regulate Akt/GSK3-β pathway activity 9,27. However, its expression level is markedly decreased in CRC due to the previous research, which provides a possibility that TRPM4 may function as a potential tumor suppressor 11. Thus, the further investigation is of urgent need.

In our study, TRPM4 was found to be frequently downregulated in CRC cell lines and cases because of CGI methylation in its promoter region and the declined expression significantly related to higher tumor stages. Meanwhile, restoring TRPM4 expression inhibits tumor cell growth both in vitro and in vivo, supporting it as a promising TSG in CRC and its methylation as a cancer-specific event. Immunohistochemistry results showed that TRPM4 expression has strong correlation with cancer metastasis, which was validated by further migration and invasion assay and tumor metastasis in vivo. Cell motility is ultimately driven by adapted cytoskeletal rearrangement whichever signaling pathway is activated 28. Due to the immunofluorescence analysis, the actin fibres were drastically lessened than mock controls in both SW48 and HT29 cell lines, indicating TRPM4 expression engage in cytoskeleton remodeling. According to these findings, the exploration of the underlying mechanism through which TRPM4 inhibits CRC migration and the possible signaling pathway are of great necessity.

Calcium homeostasis controls multiple cellular processes including proliferation, apoptosis, angiogenesis and metastasis while its dysfunction always leads to tumorigenesis 29. Numerous components of tumor migration mechanism are Ca^2+^ sensitive. Our study found that TRPM4 expression significantly increased the cytosolic Ca^2+^ level through Ca^2+^ influx in comparison with controls. The next examination of Ca^2+^-mediated signaling pathway found calpain activity and expression level were remarkably higher. Calpain proteases are known as important regulator of cytoskeleton and focal adhesion turnover during physiological cell migration. Nonetheless, excessive calpain activity in pathological phenomena may accelerate cytoskeletal proteins breakdown and promote proteolysis of FAs and proto-oncogene products 20,30.

The metastatic cascade is an intricate multistep process including several main steps including loss of intercellular contacts and invasion of the extracellular matrix (ECM) and vasculature. There are large numbers of key protein participants in this cascade 6. To invade the surrounding stroma, the speed of the formation and disassembly of the structural connection between the ECM and cytoskeleton, known as focal adhesion turnover, is crucial in tumor migratory efficiency 7. A marked component of focal adhesion system termed FAK, which has capacity to coordinate the attachment between matrix and actin cytoskeleton, was dramatically downregulated after TRPM4 expression in our study 31. The alternative explanation is due to Ca^2+^-calpain mediated proteolysis. On the other hand, degradation of ECM induced by active plasma membrane protrusions named invadopodia is of great necessity. Invadopodia can secret matrix metalloproteinases (MMPs) characterized by MMP2 and MMP9 to remodel the ECM and enhance tumor cell motility 32,33. MMPs activity can also associate with Ca^2+^ signaling 6. We also found decreased expression of MMP2 and MMP9 in TRPM4-transfected cells. E-cadherin is one of the central participants in cell-cell adhesion and the well-established marker of epithelial-to-mesenchymal transition (EMT) proteins 34. Of interest, calpain-mediated proteolysis was identified as a potential mechanism to the cleavage of full-length E-cadherin in tumor progression 35. The considerably increased E-cadherin shown in our experiments therefore makes calpain a meaningful target for therapeutic intervention in the fight against cancer migration 31.

Considering FAK is shown to be the upstream regulator of the PI3K pathway and MMPs lies downstream of Akt, we hypothesized that the anti-metastatic function of TRPM4 may be accomplished through FAK/PI3K/Akt signaling cascade 36. After Western blot analysis, we found that TRPM4 could attenuate malignant progression of CRC metastasis through interfering with PI3K/Akt signaling pathway and modulating some key proteins in metastatic cascade. The Akt family contains a serine/threonine protein kinase that is stimulated by different extracellular irritants via the PI3K pathway. Its role in tumor cell growth and survival has been well established in numerous types of cancers, of interest, multivariate studies highlight its active engagement in tumor migratory regulation 28,37. Activated Akt phosphorylates various substrates that perform different functions in tumor metastasis, involving several cytoskeleton-regulating proteins and epithelial-mesenchymal transition (EMT)-activating proteins 28. The present study showed a tremendously inhibited expression of both PI3K and Akt as well as their phosphorylated substrates (p-PI3K and p-Akt) after TRPM4 transfection. Besides, the expression of cytoskeleton-associated vimentin was significantly decreased while EMT-marker E-cadherin endowed obvious increased expression. The mammalian target of rapamycin (mTOR) is a key downstream target of Akt that has emerged as a pivotal node in many human tumors 38. So we next examined its expression and found downregulation in both mTOR and p-mTOR. Meanwhile, the expression of raptor, rictor and p-p70 were also decreased. Consequently, the tumor growth suppression may attribute at least partly to the dysfunction of mTOR network.

It was of interest to found higher expression of the protein complex consisting of the STIM1 and Orai1 in TRPM4-transfested CRC cells. STIM/Orai system is now known as key components of store-operated calcium entry (SOCE) and new player in cell migration: the former is an endoplasmic reticulum (ER) Ca^2+^ sensor while the latter localized at plasma membrane is a pore forming Ca^2+^-selective channel subunit which regulated by STIM1 25,39. Our results provide a possible explanation that the enhanced calcium influx after TRPM4 expression may at least partly due to the activation of STIM1/Orai1 protein complex and the subsequent increase of SOCE. The Rho family of small GTPases represented by Rho, Rac and Cdc42 contributes to tumor cell migration through controlling the actin cytoskeletal dynamics 40. Rac remodels the actin cytoskeleton to accelerate the formation of lamellipodia which drive tumor cell motility, and Cdc42 promotes the formation of actin-rich microspikes to induce direct cell movement 41. Our study showed the depressed expression of Rac1/2/3 and cdc42, which may partly responsible for aggression suppression of CRC.

In a word, our study provides the first evidence that TRPM4 is a novel TSG which is downregulated through epigenetic methylation in CRC. Its expression can restrain tumor proliferation as well as metastasis. The identified reorganization of cytoskeleton system and enhancement of Ca^2+^-calpain activation are highly correlated with altered migratory ability. The further studies found the metastatic suppressive role of TRPM4 is likely to be achieved by calpain-mediated FAK cleavage and inhibition ofPI3K/Akt/mTOR signaling pathway. Multitudes of other crucial proteins in metastatic cascade such as E-cadherin and MMPs are also aberrant-expressed. These results lead to better understanding of TRPM4 channel function in CRC metastasis, meanwhile, providing promising therapeutic strategies for CRC aggressiveness.

## Supplementary Material

Supplementary figures and table.

## Figures and Tables

**Figure 9:**
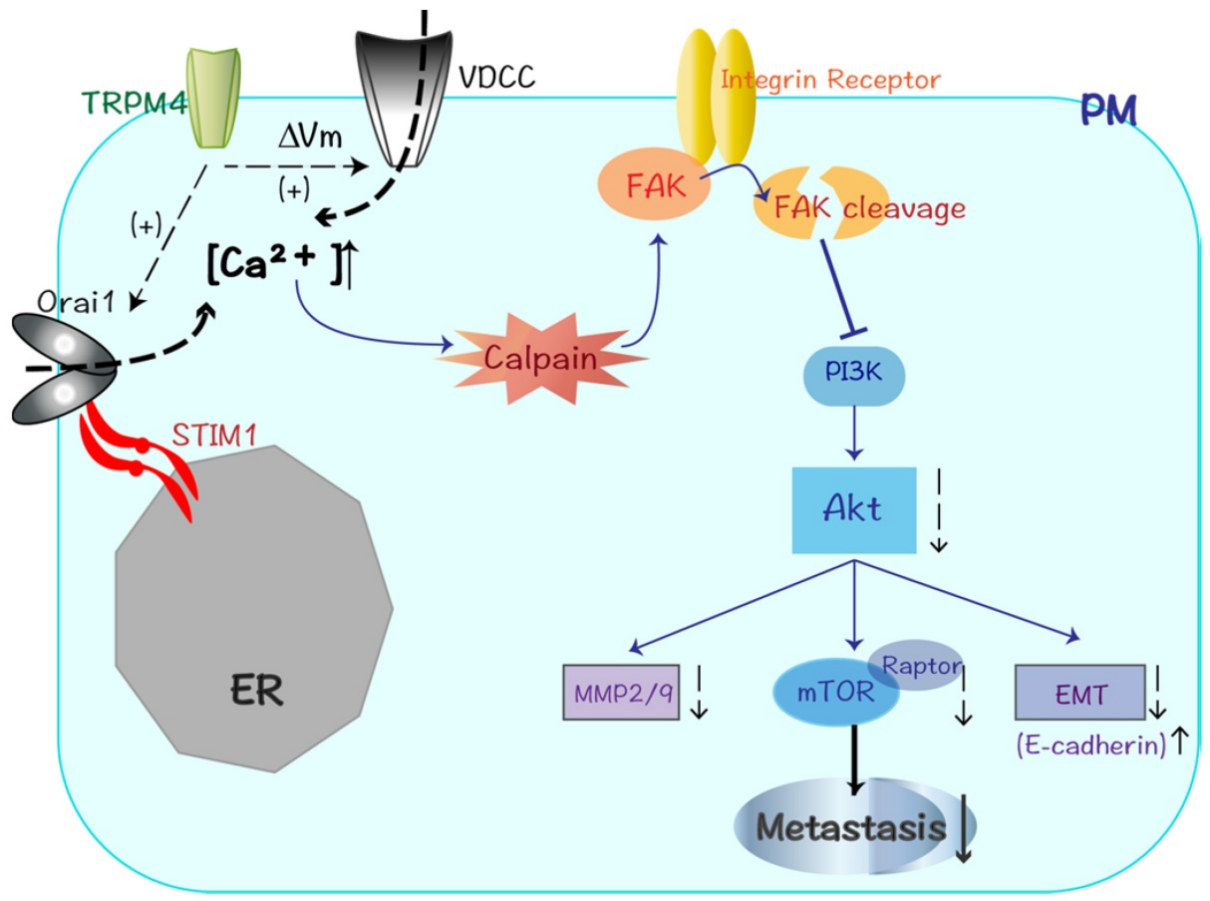
Diagram showing the mechanisms that TRPM4 expression inhibits CRC metastasis through Ca^2+^-calpain activation and PI3K/Akt/mTOR signaling pathway suppression.

**Table 1:** Association between TRPM4 expression and cilnicopathological features in CRC patients

Clinicopathological parameters	N=125	TRPM4 protein expression		*P*-value
		Low expression (n=82)	High expression (n=43)	
**Gender**				
Male	74	45	29	
Female	51	37	14	0.175
**Age**				
>62.4	65	39	26	
≤62.4	60	43	17	0.17
**Tumor location**				
Colon	77	52	25	
Rectum	48	30	18	0.565
**Differentiation**				
Well and Moderate	112	75	37	
Poor	13	7	6	0.346
**Clinical stage**				
I	20	13	7	
II	43	19	24	
III	51	39	12	
IV	11	11	0	**0.001**
**Lymph node metastasis**				
Absent	64	33	31	
Present	61	49	12	**0.008**
**Distant metastasis**				
Absent	113	70	43	
Present	12	12	0	**0.025**
**Invasion**				
T1-2	63	32	31	
T3-4	62	50	12	**0.001**

* Statistically significant (*p*<0.05)
