# Supplementary material for: A novel methylated cation channel TRPM4 inhibited colorectal cancer metastasis through Ca2+/Calpain-mediated proteolysis of FAK and suppression of PI3K/Akt/mTOR signaling pathway
Source: Int J Biol Sci. 2022 Sep 1;18(14):5575–90. doi: 10.7150/ijbs.70504 (PMC9461655; doi:10.7150/ijbs.70504)
Supplement: Supplementary file 1 — Supplementary figures and table. [file ijbsv18p5575s1.pdf]

Supplementary Figures:

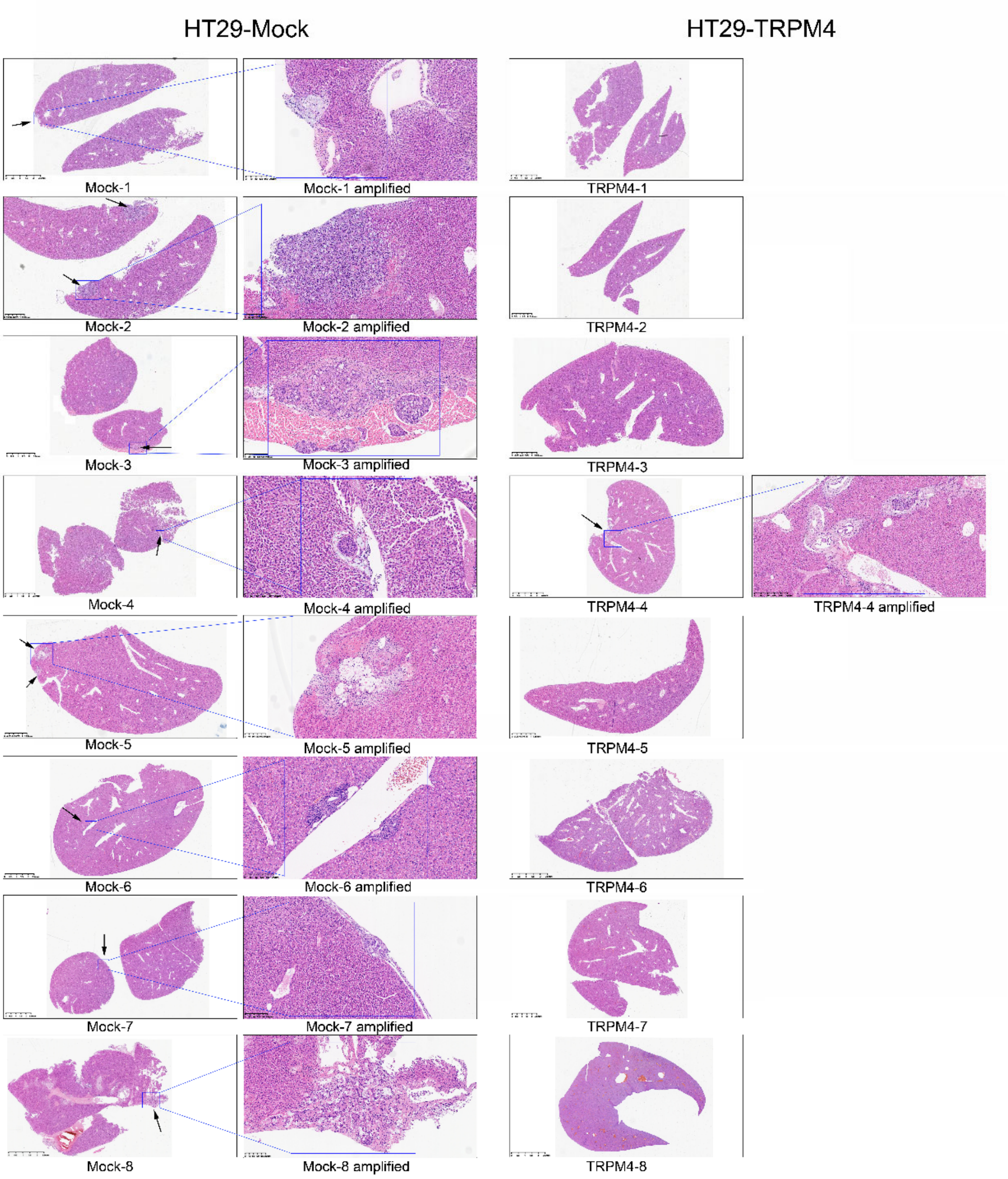

Supplementary Figure 1. The representative H&E staining of liver tissue sections of nude mice injected HT29/Mock and HT29/TRPM4 cells in abdominal cavity. The arrows indicate the metastatic nodules.

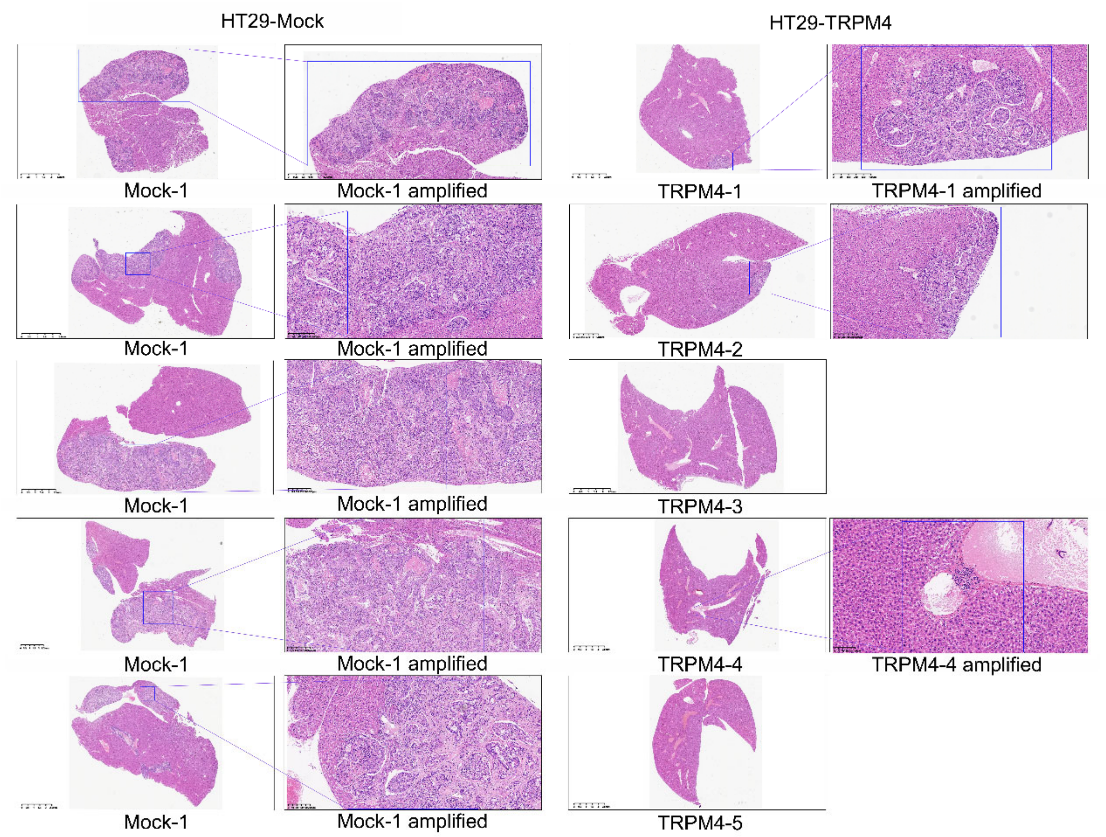

Supplementary Figure 2. Liver tissue section H&E staining images of nude mice injected HT29/Mock and HT29/TRPM4 cells in spleen. The right pictures are enlargements of the rectangle in the left pictures.

Supplementary table 1. PCR primers used in this study

**Primers**	**Sequences (5'-3')**	**Product size (bps)**
**For TRPM4 RT-PCR**		
TRPM4-F	CTGGTTCTCGCCTTCTTTTG	345
TRPM4-R	GCGTGCCAGCAGAAAGTT	
**For detecting methylated TRPM4 promoter by MSP**		
TRPM4-mF	TTTGGGTTGTAGGAGGTTGC	153
TRPM4-mR	ACCCAAAAATCTAAATATCCAACG	
**For detecting unmethylated TRPM4 promoter by MSP**		
TRPM4-uF	TTTTGGGTTGTAGGAGGTTGT	154
TRPM4-uR	ACCCAAAAATCTAAATATCCAACAC	
**For BGS analysis of TRPM4 methylation**		
TRPM4-BGS1	GATTTTTGTATTTTTTAGGT	430
TRPM4-BGS2	CTCCAAACTCAAAAATCT	
**Control for RT-PCR**		
GAPDH-F	TCCTGTGGCATCCACGAAACT	496
GAPDH-R	GAAGCATTTGCGGTGGACGAT	
